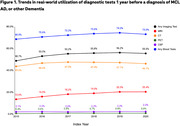# Real‐world utilization of diagnostic tests for mild cognitive impairment, Alzheimer’s disease, and other dementias in Medicare fee‐for‐service beneficiaries

**DOI:** 10.1002/alz.086946

**Published:** 2025-01-09

**Authors:** Jessie T. Yan, Allison Dillon, Tong Meng, Viviktha Ramesh, Marwan N. Sabbagh, Sophie Roth

**Affiliations:** ^1^ Roche Diagnostics, Santa Clara, CA USA; ^2^ Genesis Research Group, Hoboken, CA USA; ^3^ Roche Diagnostics, 95050, CA USA; ^4^ Barrow Neurological Institute, Phoenix, AZ USA; ^5^ Roche Diagnostics International AG, Rotkreuz, Zug Switzerland

## Abstract

**Background:**

Use of neuroimaging [e.g. magnetic resonance imaging (MRI), positron emission tomography (PET), or computed tomography (CT) scan], cerebrospinal fluid (CSF), and blood biomarker tests can contribute to a more accurate and earlier diagnosis of Alzheimer’s disease (AD). This study aimed to examine real‐world utilization of existing diagnostic tests for mild cognitive impairment (MCI), AD, or other dementias in a large US elderly population.

**Method:**

Using data from the Medicare 100% Research Identifiable files, this retrospective observational study included Medicare fee‐for‐service beneficiaries ≥ 67 years old and newly diagnosed with MCI, AD, or other dementias between 2015 and 2020. The study index date was the first disease diagnosis date. Descriptive analyses were conducted to examine the use of MRI, CT, PET, CSF, and any blood tests within 1 year before the index date in the overall study group and by three cohorts (MCI, AD, and other dementia). To understand test utilization patterns over time, test rates were also stratified by index year.

**Result:**

The final study sample consisted of 653,420 patients (9.1% MCI, 30.3% AD, and 60.6% other dementias). Overall, during the 1 year before the index date, 71.9% had at least one blood test, 53.9% had any neuroimaging test, 46.4% had CT, 17.7% had MRI, 0.7% had PET, and 2.2% had CSF. Across cohorts, the other dementia cohort had the highest utilization of any neuroimaging test (56.2% vs. 51.4% in AD vs. 46.9% in MCI). The MCI cohort had the highest utilization of CSF (3.5% vs. 2.2% in other dementias vs.1.8% in AD). The use of these diagnostic tests increased slightly from 2015 to 2020 (see Figure 1).

**Conclusion:**

In the six years prior to the availability of disease‐modifying therapies (DMTs), blood tests, MRI and CT were the predominant diagnostic tests, while the use of CSF and PET was very infrequent. Despite the modest increasing trends, substantial improvements are needed in the use of confirmatory tests, especially PET and CSF which will be necessary for increased utilization of new DMTs.